# The effects of 8 weeks of sprint interval training on repeated sprinting and specialized ability in college volleyball players

**DOI:** 10.1371/journal.pone.0327561

**Published:** 2025-07-16

**Authors:** Chao Wei, Jing An, Lin Zhou

**Affiliations:** School of Physical Education, Shandong University, Jinan, Shandong, China; Erzurum Technical University: Erzurum Teknik Universitesi, TÜRKIYE

## Abstract

**Objective:**

This study aimed to investigate the effects of 8 weeks of sprint interval training (SIT) on repeated sprinting ability and specialized performance in collegiate volleyball players.

**Methods:**

Twenty-eight male collegiate volleyball players were randomly assigned to sprint interval training (SIT, n = 14) or high-intensity interval training (HIIT, n = 14) groups. The SIT group performed all-out sprints (6 × 30m with 30s rest) twice weekly in addition to regular volleyball training, while the HIIT group underwent high-intensity interval training alongside identical regular volleyball training. Repeated sprint ability (ideal sprint time IS, total sprint time TS, and performance decrement PD), aerobic capacity (VO₂max, velocity at VO₂max [vVO₂max], ventilatory thresholds VT_1_ and VT_2_), and volleyball-specific abilities (modified agility T-test, countermovement vertical jump [CMVJ], and spike jump [SPJ]) were assessed before and after the intervention.

**Results:**

Following the intervention, the SIT group showed significantly greater improvements than the HIIT group in VO₂max (46.93 ± 4.25 vs 50.90 ± 4.17 ml/min/kg, p < 0.001), vVO₂max (14.53 ± 1.61 vs 17.03 ± 1.15 km/h, p < 0.001), VT1 (69.61 ± 4.22% vs 74.43 ± 5.25%, p < 0.001), modified agility T-test (7.85 ± 1.04 vs 6.87 ± 0.71s, p < 0.001), CMVJ (35.77 ± 3.91 vs 40.14 ± 2.82 cm, p < 0.001), and SPJ (61.20 ± 3.92 vs 65.57 ± 2.64 cm, p < 0.001). Both groups demonstrated significant improvements in IS and TS (p < 0.05), with larger effect sizes observed in the SIT group (IS: 1.060 vs 0.581; TS: 1.164 vs 0.678). No significant between-group differences were found in PD and VT2.

**Conclusion:**

Eight weeks of sprint interval training effectively enhances repeated sprinting ability, aerobic capacity, and specialized skills in collegiate volleyball players, with particularly notable improvements in vertical jumping ability and agility. These findings suggest that SIT represents an effective and time-efficient training method for improving volleyball performance.

## Introduction

Physical performance in volleyball requires a combination of aerobic and anaerobic capacities, with players needing to perform repeated high-intensity actions interspersed with brief recovery periods throughout matches [1]. The ability to maintain high-intensity efforts with minimal fatigue is crucial for successful performance in volleyball, as players must execute multiple jumps, quick directional changes, and explosive movements during extended rallies and sets. These are the specialized abilities of volleyball players [2].

High-intensity interval training (HIIT) has emerged as an effective training method for improving both aerobic and anaerobic performance parameters in various athletic populations [3]. HIIT typically involves repeated bouts of relatively intense exercise performed at 70–100% of maximal oxygen uptake (VO2max), interspersed with recovery periods [4]. A particularly intense variant of HIIT, known as sprint interval training (SIT), involves “all-out” or supramaximal efforts performed at intensities equal to or greater than 100% VO2max [5]. Recent meta-analyses have demonstrated that both HIIT and SIT can effectively improve cardiorespiratory fitness, with improvements in VO2max ranging from 3.6 to 13.6% [6]. SIT has gained particular attention due to its time-efficient nature, as it can elicit similar or greater physiological adaptations compared to traditional moderate-intensity continuous training (MICT) despite a substantially reduced training volume [7]. The effectiveness of SIT is attributed to its ability to simultaneously stress both aerobic and anaerobic energy systems [8]. A meta-analysis of SIT interventions reported moderate-to-large effect sizes for improvements in both aerobic (ES = 0.69) and anaerobic (ES = 0.61) performance measures [9]. The intense nature of SIT has been shown to enhance multiple aspects of performance, including maximal aerobic capacity, anaerobic power, and repeated sprint ability (RSA) [10].

RSA is defined as the ability to perform repeated sprints with minimal recovery between efforts, which closely mirrors the demands of volleyball competition [8]. Research has shown that SIT can improve RSA through various physiological adaptations, including enhanced phosphocreatine resynthesis, improved H+ buffering capacity, and increased oxidative enzyme activity [11]. The physiological adaptations to SIT appear to be mediated through both central and peripheral mechanisms [12]. Studies have shown that SIT can increase maximal cardiac output [13], enhance skeletal muscle oxidative capacity, and improve muscle buffer capacity [11].

In team sports specifically, RSA has been identified as a crucial fitness component [4]. For volleyball players specifically, previous studies have demonstrated that additional high-intensity training can improve agility, repeated sprint ability, and high-intensity intermittent exercise performance [2]. Recent research has also indicated that short sprint interval training is suitable for enhancing physiological and performance adaptations in volleyball players [14]. These adaptations are particularly relevant for volleyball players, who require both high-intensity burst capabilities and the ability to maintain performance throughout extended matches.

Therefore, the purpose of this study is to examine the effects of an 8-week SIT program on repeated sprinting ability and specialized ability in college volleyball players. The hypothesis of this study was 8-week SIT program can improve the repeated sprinting ability and specialized performance in college volleyball players.

## Method

### Participants

A priori power analysis was conducted using G*Power 3.1 to determine the required sample size. Based on a repeated-measures ANOVA (within-between interaction), with a medium effect size (f = 0.25), an alpha level of 0.05, and a statistical power of 0.80, the required sample size was calculated to be 28 participants (two groups × two time points: pre- and post-intervention). Accordingly, twenty-eight male collegiate volleyball players voluntarily participated in this study and provided written informed consent. Participant recruitment took place between March 6 and April 6, 2024. All participants were members of two teams competing in provincial-level tournaments and had a minimum of five years of volleyball experience. Prior to enrollment, all individuals completed a medical screening to rule out any health complications or injuries that would contraindicate participation in high-intensity exercise.

**Fig 1:**
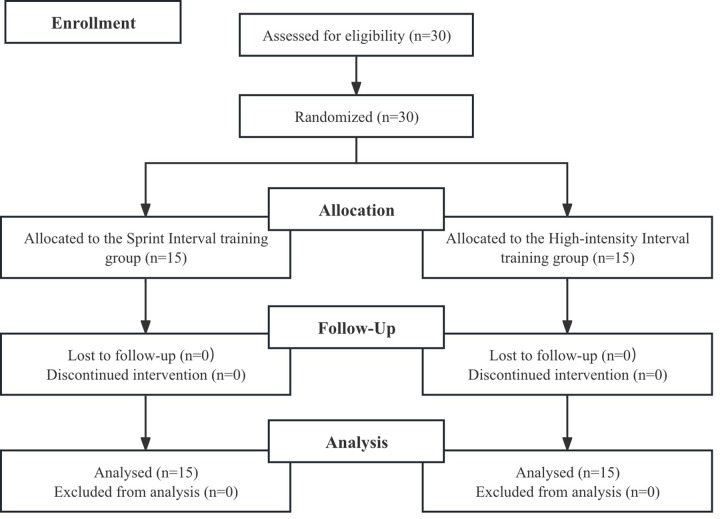
Flow chart of the progress through the phases of the study according to the CONSORT statements.

Participants were matched based on their playing positions (i.e., setter, middle blocker, hitter, and libero) and were then randomly assigned to one of two training groups: the sprint interval training (SIT) group (age = 19.8 ± 2.67 years, height = 186.2 ± 3.6 cm, body mass = 77.58 ± 6.3 kg) or the high-intensity interval training (HIIT) group (age = 20.7 ± 1.4 years, height = 186.9 ± 3.2 cm, body mass = 76.4 ± 3.3 kg). All participants were familiar with all-out interval training but had not engaged in any structured HIIT program in the three months preceding the study (see Fig 1).

The study was approved by the Shandong University institutional Ethics and Research Committee (protocol number: IRB-SBMS-SDU(S)2025-01-79), followed the ethical principles contained in the Declaration of Helsinki. All players participated voluntarily in this study and a written consent form was signed before entering the study.

### Procedures

The study employed a randomized controlled trial with two experimental groups. Participants were first matched according to playing position (i.e., setter, middle blocker, hitter, and libero), then randomly assigned to the SIT or HIIT groups. Group allocation was conducted using a computerized random number generator by an independent researcher who was not involved in either training delivery or outcome assessments. To ensure allocation concealment and assignment unpredictability, group assignments were placed in opaque sealed envelopes and opened only after the matching process. Prior to initiating the study, participants underwent a series of assessments encompassing laboratory and field-based measurements. These evaluations aimed to gauge cardiorespiratory fitness indicators and volleyball-specific motor abilities. Participants underwent a graded exercise test on the first visit to evaluate maximum oxygen uptake (V̇O_2max_) and related physiological variables. The second visit encompassed the assessment of repeat sprint test. Jumping ability was measured during the third testing session, while the fourth occasion involved evaluating change of direction (COD). There was a 24-hour recovery period between testing sessions, during which participants were directed to refrain from alcohol and caffeine. Additionally, they were directed to abstain from engaging in vigorous physical activity during this timeframe. Subjects were also asked to complete food diaries 3 days before baseline testing and to replicate this diet before the post-training test session. ~ 48 hours after the last testing session, participants began the 8-week training program. Subsequently, 48 hours after the final training session, they underwent the same testing procedure, following the identical order and under the same conditions as the pre-test. Due to the nature of the intervention and the group-based training setting, blinding of participants and intervention supervisors was not feasible. However, outcome assessments were conducted using automated or objective tools, such as electronic timing gates and gas analyzers. While assessors were not blinded to group allocation during data collection, test protocols and procedures were standardized, and instructions were strictly followed to minimize bias (Table 1).

### Graded Exercise Test

Aerobic fitness was evaluated using a graded exercise test [15].Following a 10-minute warm-up comprising five minutes of jogging and five minutes of dynamic stretching, athletes performed the graded exercise test on a treadmill (Technogym, Cesena, Italy). The test started at an initial intensity of 8 km·h^–1^, with velocity increasing by 1 km·h^–1^ every three minutes. Each stage had 30 seconds of relief intervals, during which blood lactate concentration was measured through earlobe blood sampling. A breath-by-breath gas collection system (MetaLyzer 3B-R2, Cortex, Germany) continuously recorded cardiorespiratory fitness measures throughout the test. The device measured V̇O_2max_, as well as the first and second ventilatory thresholds (VT_1_ and VT_2_), following standard criteria [16]. The minimal velocity that V̇O_2max_ elicited was established as vV̇O_2max_ if it could be maintained for a minimum of one minute. Two independent researchers localized the first and second ventilatory thresholds (VT_1_ and VT_2_). The VT2 identification criterion was a continuous rise in the V̇_E_ equivalent for O2 (V̇E V̇O_2_^−1^) and the V̇_E_ equivalent for CO2 (V̇E V̇CO_2_^−1^) ratio curves in relation to the decrease in end-tidal O_2_ tension (P_ET_O_2_). The first ventilatory threshold (VT1) was also established as the point where an increase in V̇E V̇O_2_^−1^ and P_ET_O2 occurred without a simultaneous rise in the V̇E V̇CO_2_^−^1 [16].

### Repeated Sprint Test

The RST protocol included a series of six 30-m maximal sprints with 30-second rest periods between runs. A photoelectric cell timing system (Alge-Timing Electronic, Vienna, Austria) linked to a digital chronoscope was used to record each sprint and rest interval time, with an accuracy of 0.001 seconds. During the recovery period between sprints, participants tapered down from the sprint they had just completed and walked back to the next starting point. Two sets of timing gates were used, working in opposite directions, to allow participants to start the next run from the ending point of the preceding sprint, thus eliminating the need to hurry back to the initial starting point. A standing start, with the front foot placed 30 cm behind the timing lights, was used for all sprints. Timing was initiated when the participant broke the light beam. An experimenter was placed at each end of the track to provide strong verbal encouragement to the participants at each sprint.

The three performance metrics derived from the RST were: (1) *Ideal Sprint time (IS)*, calculated by multiplying the fastest 30-m sprint time by six; (2) *Total Sprint time (TS)*, calculated as the sum of all six sprint times; and (3) *Performance Decrement (PD)*, calculated as ([TS/IS] × 100) – 100, representing fatigue across sprints [17]. The test-retest reliability of running RST is 0.942 for TS and 0.75 for PD [17,18].

### Modified agility T-Test

**Fig 2:**
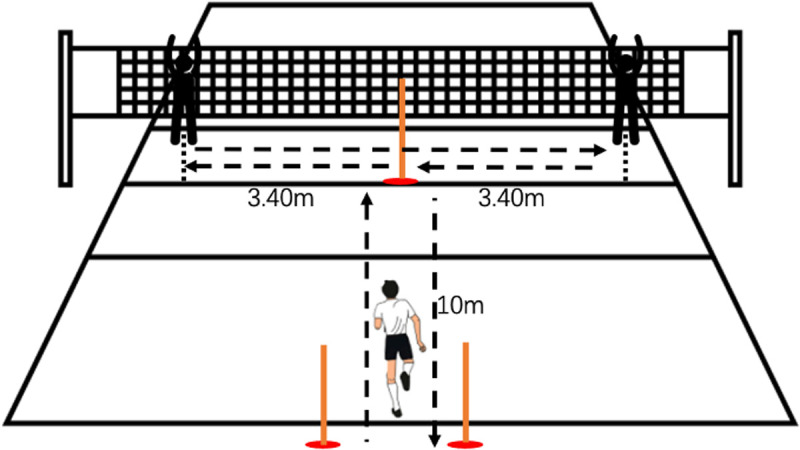
Modified agility T-Test.

Change of Direction (COD) performance was evaluated using the modified agility T-test (MAT)(Fig 2), a modified version of the T-test, including elements specific to the court tasks of volleyball players [19]. The test was modified to create additional volleyball-specific situations. The forward and backward running distance was extended to 10 m. Instead of lateral shuffling to cones, participants were instructed to perform standardized volleyball jump blocks on both sides of the net using both hands raised above the head, ensuring consistency across all trials.

Each player started on the court axis, one meter behind the end line, sprinted forward 10 meters, and touched a central reference pole placed on the centerline of the court with both hands. The player then moved to either the left or right side and executed a jump block targeting a vertical pole positioned above the net, ensuring that the pole was placed between the player’s hands during the block. After completing the first block, the player returned to the central pole, then repeated the same jump block on the opposite side. Finally, the player returned again to the central pole, touched it with both hands, and backpedaled to the starting position.

The MAT was timed manually using a digital stopwatch operated by the same trained assessor. Each participant completed three trials, and the fastest time was recorded. The intra-rater reliability (ICC) for manual MAT timing in our pilot testing was 0.93. The test duration ranged from 10 to 20 seconds depending on the player’s speed.

### Jumping ability

A wall-mounted jump tester (VERTEC Power System, USA) was utilized on the volleyball court to measure the countermovement vertical jump (CMVJ), spike jump (SPJ). To assess CMVJ, the participants were directed to perform vertical jumps with maximum effort, to determine their jump height in centimeters. This involved executing a countermovement knee flexion until reaching a 90° angle which marked with elastic band while refraining from using their arms. The examination commenced with the participants standing, followed by knee flexion and then jumping as high as they could possibly achieve [20]. The reliability coefficient (ICC) for repeated measurements at CMJ was 0.95. The SPJ began with the assessment of the participant’s standing reach height using the VERTEC device mentioned earlier. Subsequently, the participant was instructed to position themselves on the floor, maintaining 3–4 m from the VERTEC device. Upon receiving the investigator’s command, the participants initiated an approach run toward the VERTEC device and executed a complete spiking action using the palm of their spiking hand. A standard run-up for a volleyball spike, involving four-step approaches, was permitted. The score for the SPJ was recorded by subtracting the jumping height from the participant’s standing reach height in centimeters [21]. The ICC for repeated measurements at SPJ was 0.93. Each test was performed three times, and the best score was selected for further analysis. Each participant performed 3 maximal jumps for each test, with a 30-sec rest period between each attempt. The coefficient of variation (CV) of the CMVJ and SPJ were 2.24% and 3.13%, respectively.

### Training program

Training sessions started with ~15 min of skill-based volleyball drills warm-up, jogging, full-body stretching, and submaximal jumps. The SIT group performed two sessions per week for four weeks, each session including 2 sets of 6 maximal 30-meter sprints, with 30 seconds of passive recovery between repetitions and 3 minutes between sets. All sessions were supervised by strength and conditioning coaches, and sprint performance was recorded to ensure maximal effort.

The HIIT group underwent their regular volleyball skill-based training sessions and additionally performed a HIIT protocol twice per week for 4 weeks. The HIIT program consisted of 2 sets of 6 bouts of 20-second high-intensity running efforts at 90–95% HRmax, with 30 seconds of passive recovery between bouts and 3 minutes of rest between sets. All sessions were performed on an indoor volleyball court to simulate sport-specific movement patterns. Players ran shuttle sprints between two cones placed 10 meters apart, emphasizing rapid acceleration and change of direction. The total training volume and intensity were matched as closely as possible to the SIT group.

### Statistical analyses

The experimental data were processed by JASP (version 0.18.3, JASP team, The Netherlands). Data were expressed as mean ± standard deviation (M ± SD). The normality of the data was assessed by the Shapiro-Wilk test. If the data was normally distributed, the differences in the outcomes at baseline were examined using one-way ANOVA. If the data was normally distributed, we used two-way repeated measure ANOVA to examine the effects. The dependent variable for each model was each of the primary outcomes. The model effects were group (SIT vs. HIIT), time (pre- and post- intervention), and their interaction. To validate the use of repeated-measures ANOVA, we checked the assumptions of sphericity and homogeneity of variances. Sphericity was assessed using Mauchly’s test, and if violated, the Greenhouse-Geisser correction was applied. Homogeneity of variances was assessed using Levene’s test. When a signiﬁcant interaction was observed, and Bonferroni post-hoc comparison was performed to identify where the signiﬁcance was. Cohen’s d (d) values were used to assess the effect size and 95% confidence intervals for the effect sizes, and it was classiﬁed as trivial (d < 0.2), small (0.2 ≤ d ≤ 0.6), moderate (0.6 ≤ d ≤ 1.2), large (1.2 ≤ d ≤ 2.0), or very large (d > 2.0) [22]. The signiﬁcance level of these models was set at p < 0.05.

## Results

All the participants completed this study, and all the data were included in the analysis. No significant difference in the outcomes measured were observed between both group (P > 0.413).

**Table 1:** The main part of the training session for repeated sprint training group and high-intensity interval training group.

Exercise	The first stage (week 1-2)	The second stage (week 3-5)	The third stage (week6-8)
Sprint training (twice a week)	Intensity: 30-m all-out sprints Volume: 6 rep/series,2 series Rest: 30sec/rep,2min/series	Intensity: 30-m all-out sprints Volume: 6 rep/series,3 series Rest: 30sec/rep,2min/series	Intensity: 30-m all-out sprints Volume: 6 rep/series,2 series Rest: 30sec/rep,2min/series
High-intensity intermittent training (twice a week)	Intensity: 90% HRmax Volume: 20sec*6rep/series,2 series Rest: 30sec/rep,2min/series	Intensity: 90% HRmax Volume: 20sec*6rep/series,3 series Rest: 30sec/rep,2min/series	Intensity: 90% HRmax Volume: 20sec*6rep/series,2 series Rest: 30sec/rep,2min/series

Sec: second; V_YIR1T:_ velocity achieved during the final completed stage at the Yo-Yo IR1 Test

**Fig 3:**
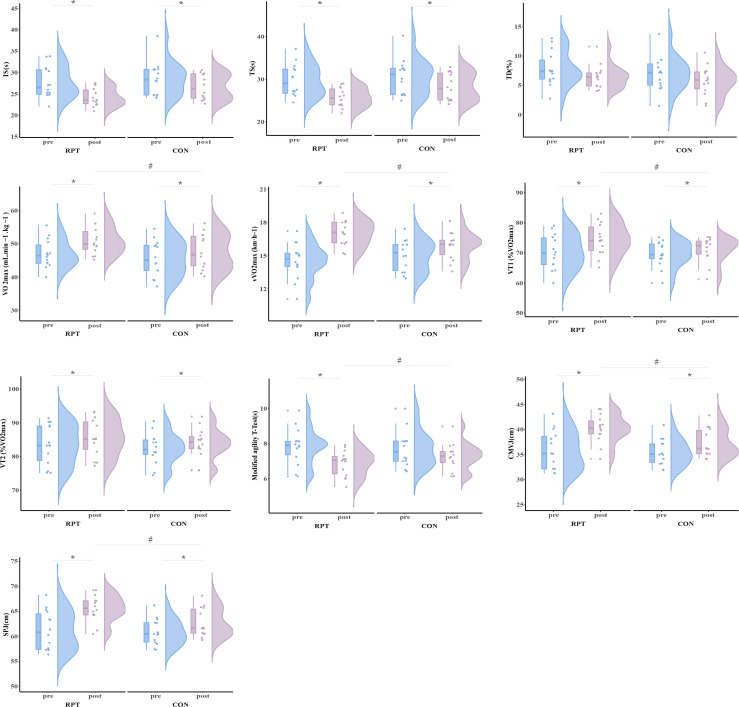
The strength and performance before and after Training. * Statistically signiﬁcant difference between pre-and post-test, p < 0.05.# Statistically signiﬁcant difference between group, p < 0.05.

The primary two-way repeated-measures ANOVA models showed significant main effects of time, and interactions between group and time on VO_2max_ (P < 0.001), vVO_2max_ (P < 0.001), VT1 (P < 0.001), Modified agility T-Test (P = 0.041), CMVJ (P = 0.003), and SPJ (P = 0.003). The post-hoc analysis revealed that VO_2max_ (t = 18.045, P < 0.001), vVO_2max_ (t = 8.614, P < 0.001), VT1 (t = 10.844, P < 0.001), modified agility T-Test (t = 5.461, P < 0.001), CMVJ (t = 7.792, P = 0.001), and SPJ (t = 7.794, P = 0.001) in SIT group were significantly greater after training as compare to HIIT group. Within HIIT group, VO_2max_ (P < 0.001), vVO_2max_ (P = 0.049), VT1 (P = 0.004), CMVJ (P = 0.020), and SPJ (P = 0.020) were significantly greater after training as compared to baseline(Table 2 and Fig 3).

**Table 2:** The assessment results for SIT group and HIIT group before and after 8-week training.

Variable		SIT (N=14)			HIIT (N=14)		
		Pre	Post	Cohen’s d (95% CI)	Pre	Post	Cohen’s d
RSA Test	IS (s)	27.60±3.65	24.16±2.11^*^	1.060 (0.386, 1.734)	28.50±3.94	26.62±2.95^*^	0.581 (0.001, 1.160)
	TS (s)	29.71±3.75	25.72±2.24^*^	1.164 (0.413, 1.914)	30.48±4.21	28.15±3.21^*^	0.678 (0.023, 1.333)
	PD (%)	7.78±2.91	6.44±2.01	0.515 (-0.461, 1.492)	6.97±2.93	5.77±2.48	0.460 (-0.512, 1.433)
VO_2max_ (ml/min/kg)		46.93±4.25	50.90±4.17^*#^	0.827 (0.475, 1.179)	45.55±5.34	47.53±5.33^*^	0.413 (0.204, 0.622)
vVO_2max_ (km/h)		14.53±1.61	17.03±1.15^*#^	1.842 (0.911, 2.773)	15.05±1.41	15.89±1.23^*^	0.613 (-0.030, 1.256)
VT1 (%)		70.15±5.78	74.43±5.25^*#^	0.875 (0.460, 1.290)	69.61±4.22	71.14±4.12^*^	0.314 (0.053, 0.575)
VT2 (%)		83.46±5.88	85.51±5.46^*^	0.400 (0.174, 0.626)	82.29±4.51	83.85±4.48	0.306 (0.105, 0.508)
Modified agility T-Test (s)		7.85±1.04	6.87±0.71^*#^	1.086 (0.387, 1.785)	7.68±1.02	7.25±0.77	0.480 (-0.107, 1.067)
CMVJ (cm)		35.77±3.91	40.14±2.82^*#^	1.424 (0.668, 2.179)	35.43±2.59	37.24±2.78^*^	0.591 (0.029, 1.154)
SPJ (cm)		61.20±3.92	65.57±2.64^*#^	1.438 (0.675, 2.201)	60.84±2.60	62.65±2.80^*^	0.597 (0.029, 1.165)

* Statistically signiﬁcant difference between pre-and post-test, p < 0.05;

# Statistically signiﬁcant difference between group, p < 0.05.

The primary two-way repeated-measures ANOVA models showed significant main effects of time but no interactions between group and time on IS (P = 0.086), TS (P = 0.122), TD (P = 0.912), and VT2 (P = 0.250). Within both SIT and HIIT group, IS (SIT: P < 0.001; HIIT: P = 0.031), and TS (SIT: P < 0.001; HIIT: P = 0.024) were significantly greater after training as compared to baseline(Table 2 and Fig 3).

## Discussion

This study aimed to evaluate the effects of an 8-week SIT program on RSA, aerobic capacity, and volleyball-specific performance among collegiate male players. The findings confirm that SIT not only improved general physiological indicators but also effectively enhanced performance in explosive and agility-related volleyball tasks.

A key finding of this study was that both the SIT and HIIT groups exhibited significant within-group improvements in ideal sprint time (IS) and total sprint time (TS) following the 8-week intervention. Notably, the SIT group demonstrated larger effect sizes (IS: d = 1.060; TS: d = 1.164) compared to the HIIT group (IS: d = 0.581; TS: d = 0.678), suggesting that SIT may induce more substantial improvements in sprint execution. These changes occurred despite the absence of significant group-by-time interactions or notable reductions in performance decrement (PD), indicating that the benefits of SIT were more pronounced in enhancing sprint quality than in mitigating fatigue accumulation. These findings are consistent with previous studies. For example, Kelly [23] and McGawley and Taylor [24] both reported similar improvements in sprint performance, particularly in terms of maintaining higher sprint intensities. However, in contrast to those studies, our protocol did not yield significant improvements in PD, possibly due to the relatively long rest intervals (30 s) between repetitions, which may have attenuated fatigue development. These intervals may have allowed for more complete recovery between sprints, thereby reducing the cumulative fatigue that typically contributes to performance decline. This was supported by Robineau [25], which showed that SIT could significantly enhance the mean performance of RSA instead of PD. Moreover, the larger effect size in IS and TS observed in the experimental group may indicate that SIT’s impact is more pronounced in improving the quality of individual sprints (i.e., sprint intensity and total sprint time) rather than endurance across multiple sprints. This is supported by the idea that SIT enhances neuromuscular adaptations, particularly in fast-twitch muscle fibers [26], which are crucial for explosive movements such as sprints. These adaptations likely led to greater improvements in sprint intensity and total sprint time in the experimental group.

Regarding volleyball-specific abilities, the SIT group achieved significantly greater improvements than the HIIT group in countermovement vertical jump (CMVJ), spike jump (SPJ), and modified agility T-test performance, as indicated by large effect sizes (CMVJ: d = 1.424; SPJ: d = 1.438; agility test: d = 1.086). These improvements are highly relevant to volleyball, where vertical power and rapid directional changes are essential for successful blocking, spiking, and court coverage. While the HIIT group also showed moderate gains in CMVJ and SPJ, the superior outcomes in the SIT group suggest a more potent stimulus for developing explosive capabilities. The enhancement in agility performance further demonstrates SIT’s capacity to improve dynamic movement efficiency even under fatigue. These findings provide strong evidence that SIT has a targeted effect on volleyball-specific movements, such as vertical jumping and agility. The results of this study align with those of Song [27]who observed similar improvements in jump height and athletic performance following SIT. Fang and Jiang [28]also found that SIT enhances explosive power and performance in both male and female athletes, especially in sports requiring rapid, high-intensity movements. Additionally, the results of modified agility T-test in this study reflects the ability to maintain power and agility post-fatigue. These improvements in this test highlight that SIT not only enhances jump height but also the capacity to perform explosive movements after intense exertion, which represents a significant contribution of this study.

In addition to the improvements observed in repeated sprinting performance and volleyball-specific skills, the study also found SIT achieved significantly greater improvements in key physiological indicators compared to HIIT, including VO2max, vVO2max, VT1. The increase in VO2max and vVO2max is in line with findings from previous studies such as those by Sloth [29] and de Oliveira-Nunes [30], who reported that SIT can enhance both maximal oxygen uptake and the ability to perform at higher intensities. The observed improvements in VT1 and VT2 within the SIT group further support these findings, indicating an enhanced ability to maintain aerobic performance at submaximal and maximal intensities. However, the lack of significant between-group differences in VT2 suggests that the SIT may not have had a unique or consistent effect on this threshold. This finding contrasts with study like Liu [31], which found that SIT could significantly improve VT2 and overall endurance performance. The discrepancy between these studies and ours may be attributed to differences in SIT protocols, such as the rest intervals. In our study, the longer rest periods between sprints may have allowed for more complete recovery, which could reduce the cumulative fatigue that typically contributes to a significant reduction in performance at higher intensities. SIT offers significant benefits for college physical education due to its time-efficiency and effectiveness. It provides a practical solution for time-constrained college students while significantly enhancing volleyball-specific abilities and fundamental fitness indicators including aerobic capacity, agility, and explosive power. The simplicity of SIT protocols makes them easily implementable in college settings without specialized equipment, benefiting both dedicated student-athletes and non-sports majors on school teams who need to balance athletic development with academic responsibilities. Additionally, the efficacy of high-intensity interval training protocols has been demonstrated not only in athletic populations but also in rehabilitation settings. For instance, a study on individuals post-ACL reconstruction reported significant improvements in muscle strength and aerobic capacity following a HIIT regimen [32]. These findings align with our results, suggesting the broad applicability of HIIT in enhancing physical performance.

However, several limitations of this study should be acknowledged. First, we did not monitor the training load for either group. Future research should consider tracking the Training Impulse (TRIMP) or other relevant training load metrics to provide a more accurate comparison of the intensity and volume between the two training regimens. This would offer deeper insights into the differences in training effects and help clarify how SIT compares with traditional training in terms of workload and recovery. Second, we did not include biochemical analyses such as lactate, hemoglobin, or other relevant blood markers. which could have provided a more comprehensive understanding of how SIT affects volleyball players’ physiological responses and athletic performance. Third, the performance testing sessions followed a fixed order (GXT, RST, Jumping, COD) over consecutive days, with 24-hour recovery between tests. Although recovery time was standardized, cumulative fatigue or learning effects across multiple days cannot be entirely ruled out. Future studies should consider counterbalancing the order of tests to reduce potential order effects. Fourth, assessors were not blinded to group allocation during the performance tests (RST, MAT, Jumps), which could introduce observer bias. Future studies should incorporate blinded assessors to strengthen the objectivity of test outcomes. Finally, future research may also explore how variations in SIT protocols and their integration with sport-specific training influence long-term skill retention, recovery dynamics, and injury prevention in volleyball players.

## Conclusion

Our study demonstrates that 8 weeks of SIT significantly improves repeated sprinting performance and volleyball-specific skills, highlighting its effectiveness for athletes in high-intensity sports. These findings suggest that SIT is a promising, time-efficient method for enhancing both anaerobic and aerobic performance. For college physical education, SIT provides a time-efficient, easily implemented, and significantly effective training method that is applicable not only for specialized volleyball training but also as an effective means to improve the overall physical fitness of college students, making it worthy of broader application in college physical education curricula and campus sports team training.
